# Design Principles for Next Generation of Small Organic Molecules for Photodynamics Therapy Revealed by Nonadiabatic Molecular Dynamics

**DOI:** 10.1002/chem.70941

**Published:** 2026-03-30

**Authors:** Vinícius N. da Rocha, Davide Avagliano, Paulo C. Piquini

**Affiliations:** ^1^ Department of Physics Federal University of Santa Maria Santa Maria RS Brazil; ^2^ Institute of Chemistry for Life and Health Sciences (iCLeHS, UMR 8060) Chimie ParisTech, PSL University, CNRS Paris France

**Keywords:** intersystem crossing, molecular design, nonadiabatic dynamics, photodynamics therapy, surface‐hopping

## Abstract

Nitrobenzochalcogenadiazole derivatives are emerging candidates for photodynamic therapy (PDT), yet the precise mechanisms governing their excited‐state deactivation and triplet generation remain to be fully elucidated. This study employs nonadiabatic dynamics simulations, using the trajectory surface hopping method within a linear vibronic coupling (LVC) framework, to unravel the intersystem crossing (ISC) pathways in these systems. We systematically investigate two design factors: the heavy‐atom effect (substituting S with Se and Te) and the influence of “push–pull” electronic architectures (donor–acceptor vs. donor–donor/acceptor–acceptor). Our results demonstrate that replacing sulfur with selenium and tellurium monotonically accelerates ISC, reducing excited‐state lifetimes from ≈ 6.1 ps to sub‐picosecond timescales (≈ 0.9 ps) via a dominant $S_{2}\to S_{1}\to T_{n}$ relaxation channel driven by enhanced spin–orbit coupling. Furthermore, we reveal that structural modifications that disrupt this push–pull nature (D–D or A–A) result in kinetic bottlenecks, trapping the population in the singlet manifold. These dynamical insights establish clear structure–property relationships, guiding the rational design of photosensitizers with optimized triplet quantum yields.

## Graphical abstract

Molecular design reshapes the landscape of excited‐state relaxation. Nonadiabatic dynamics reveal how electronic structure guides population flow between competing pathways beyond static energetic arguments.

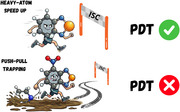

## 1 Introduction

Photodynamics therapy (PDT) is a modern, noninvasive treatment approach that has been effectively employed in managing both cancerous and noncancerous conditions. Its proven efficacy and compatibility with other therapeutic strategies have driven its expanding use in diverse medical areas such as dermatology, oncology, gynecology, and urology [1, 2].

The principle of PDT involves the administration of a photosensitizer, a compound that selectively accumulates in pathological tissues. Upon irradiation with light of an appropriate wavelength, the photosensitizer absorbs photons and undergoes a cascade of photophysical and photochemical processes. Following excitation to a singlet state, an efficient intersystem crossing (ISC) to the corresponding triplet manifold is required, enabling interaction with ground‐state molecular oxygen (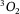). This interaction leads to the formation of singlet oxygen (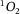) and other reactive oxygen species (ROS) [3], which are responsible for the selective destruction of target cells [4, 5]. An ideal photosensitizer must exhibit a high quantum yield of triplet‐state formation and singlet oxygen generation, chemical and photochemical stability, and preferential accumulation in diseased tissue [6]. Tetrapyrrolic macrocycles (such as porphyrins, chlorins, and corroles) and BODIPY derivatives are currently among the most utilized molecular platforms for the development of photosensitizers [7, 8, 9].

Recent studies demonstrate that efficient ISC in PDT photosensitizers can be achieved through precise control of molecular electronic structure. Donor–acceptor chromophores promote ISC by stabilizing charge‐transfer excited states, enabling efficient triplet formation even in weak spin–orbit coupling regimes [10]. Strong acceptor units, including nitro‐ and chalcogen‐containing motifs, further modulate ISC controlling excited‐state character, offering a versatile design strategy for organic photosensitizers and providing new insights into the design of metal‐free PSs [11, 12]. Importantly, application‐oriented studies confirm that such molecular‐level control directly translates into improved PDT performance [13, 14]. In this context, the exploration of metal‐free molecular architectures highlights the importance of structural diversity in the rational design of new PDT photosensitizers.

Nitrobenzoxadiazole (NBD) derivatives, small organic molecules constructed from the benzoxadiazole (BD) backbone, have been widely employed in biological studies due to their high environmental sensitivity, excellent biocompatibility, small molecular size, and charge neutrality [15, 16]. The structure of NBD derivatives can be characterized by an electron push–pull system, created by the combination of electron‐donating groups and electron‐withdrawing groups ($SO_{3}$ and $NO_{2}$, for example). The donor–acceptor (D–A) architecture facilitates intramolecular charge transfer (ICT) [17]. This enables the tuning of absorption and emission wavelengths simply by altering the electron‐donating substituents [18, 19]. Although some NBD derivatives have been reported as singlet oxygen generators, the photodynamic process of the pathways of ISC of these molecules remained limited, thereby constraining the rational design of efficient photosensitizers [15, 17, 20].

More recently, nitrobenzothidiazole (NBTD) and nitrobenzoselenadiazoles (NBSDs), sulfur and selenium‐substituted analogs of NBD, have emerged as promising photosensitizers for PDT, overcoming several limitations of conventional NBD‐based compounds as excitation wavelengths in the ultraviolet (UV) region, below 400 nm [16, 21]. The presence of the selenium atom in the NBSD core induces a redshift in the absorption spectrum, further improving light harvesting in the therapeutic window, and enhances intersystem crossing via spin–orbit coupling, promoting an efficient transition from the excited singlet to the triplet state, which is essential for the generation of ROS [17, 22, 23].

**FIGURE 1:**
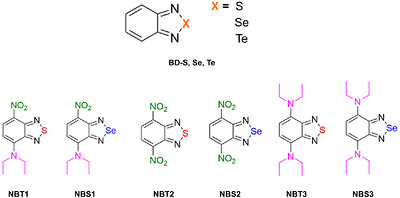
Molecular structure of benzo‐thiadiazol, selenadiazol, and nitrobenzo‐thiadiazole and selenadiazole derivatives.

The nitrobenzoselenadiazole derivative NBS1 (Figure 1), which contains a tertiary amine donor group, illustrates a mechanistic gap in the evaluation of photosensitizers [16]. This class of compounds features a characteristic ICT “push–pull” framework, in which the nitro group acts as a strong electron acceptor and the amine serves as the electron donor, enabling tunable electronic and photophysical properties. Although NBS1 belongs to this family, it exhibited high phototoxicity in U87MG glioblastoma cells while its ROS generation efficiency remains unreported, a behavior often associated with tertiary amine substituents that can hinder water coordination and reduce PDT efficiency [16]. The apparent mismatch between strong phototoxicity and uncertain ROS production highlights the need for further investigation into how donor‐group modifications influence the PDT performance of NBS1.

Computational approaches allow the investigation of the electronic structure and excited‐state dynamics of photosensitizers, providing valuable insights into the key photophysical processes that govern their efficiency, such as ISC. By analyzing how structural modifications, such as the introduction of electron‐donating or electron‐withdrawing substituents [9, 24], or coordination of heavier atoms—affect the electronic coupling between singlet and triplet states [25, 26], it is possible to predict and rationalize trends in spin–orbit coupling (SOC) and ISC rates. These theoretical predictions, when complemented by experimental photophysical characterization, enable a deeper understanding of the mechanisms that control triplet‐state population and singlet oxygen generation, thus guiding the molecular engineering of more efficient and selective photosensitizers for medical applications [27, 28, 29].

Molecular design has long been the cornerstone of progress in PDT, traditionally guided by static structure–property relationships such as energy level alignment, singlet–triplet energy gaps, and the modulation of electronic structure through the incorporation of heteroatoms and different ligands [26, 30, 31]. While these descriptors remain essential, it has become increasingly clear that the ultimate performance of a photosensitizer is determined by its excited‐state relaxation pathways [32, 33, 34], which are inherently dynamical in nature. Despite all approximations inherent to ab initio simulations of complex molecular systems, there are now cases in which both adiabatic and nonadiabatic molecular dynamics reach a level of accuracy sufficient to guide not only the interpretation of experiments, but also the rational design of new materials [35]. In this framework, excited‐state dynamics provides direct access to the mechanistic origin of intersystem crossing, population trapping, and kinetic bottlenecks, revealing which molecular motifs and nuclear motions actively control triplet‐state formation [36, 37].

Two main aspects are systematically explored in this work: (i) the influence of heteroatom substitution in the benzothiadiazole core (S, Se, and Te) to elucidate the heavy‐atom effect on ISC efficiency, and (ii) the impact of electron‐donating and ‐withdrawing substituents on the conjugated ring to assess how push–pull electronic effects modulate the excited‐state dynamics and photophysical properties.

Using nonadiabatic excited‐state dynamics, we show that efficient triplet‐state formation in nitrobenzochalcogenadiazole derivatives (Figure 1) arises from a subtle interplay between heavy‐atom effects and D–A orbital topology, providing key guidelines for the rational design of PDT photosensitizers.

## 2 Computational Methods

### 2.1 Electronic Structure Calculations

The ground‐state equilibrium geometries, vibrational frequencies, excitation energies, and gradients were computed using density functional theory (DFT) as implemented in the ORCA quantum chemistry package [38]. The wB97X‐D3 functional, which includes long‐range exchange and empirical dispersion corrections (D3 version), was used in combination with the def2‐SVP basis set. This level of theory provides a balanced description of noncovalent interactions and excited‐state potential energy surfaces while remaining computationally feasible for medium‐sized systems such as NBSeD and NBSD. The vibrational normal modes and frequencies obtained at this level were later used to sample initial conditions and momenta to initialize the dynamics in the excited states using a Wigner probability distribution [39, 40]. To further investigate the influence of heavy atom effects on ISC, an additional calculation was performed using a tellurium (Te)‐containing analogue, replacing the chalcogen atom in the BD core. To properly account for the relativistic effects associated with Te, the x2c‐TZVPall [41] basis set was employed. Excited states energies, gradient, and couplings were used to build a linear vibronic coupling (LVC) Hamiltonian that will be used to calculate the electronic energies along the dynamics [42].

## 3 Trajectory Surface Hopping Simulations

The nonadiabatic dynamics of NBSD and NBSeD were simulated using trajectory surface hopping as implemented in SHARC (Surface Hopping including ARbitrary Coupling [43]). The dynamics are propagated following excited‐state potential energy surfaces (PES) calculated on the fly via the LVC Hamiltonian we built [44]. Using an LVC model allows us to perform ps long dynamics, necessary to properly capture ISC, for a high number of compounds investigated with a reasonable computation time. The accuracy of this approximation was benchmarked by comparing the dynamics with selected initial conditions repeated with TD‐DFT (see Section S6). Trajectories were set up using a set of 500 initial coordinates and momenta sampled from a Wigner distribution of the ground‐state PES. During the dynamics, we allowed hops between three singlets and three triplets. Each of the 500 trajectories was propagated for a total simulation time of 5000 fs with a time step of 0.5 fs, yielding a statistically significant picture of the ISC pathways. An energy‐based decoherence correction was applied with a constant value of C=0.1 a.u. [45]. During surface hops, the total energy was conserved by rescaling the full velocity vector. Time derivative couplings were computed with the local diabatization scheme, approximating nonadiabatic coupling with wavefunction overlaps at consecutive steps. [46]. The nonadiabatic dynamics were performed using SHARC 3.0 [43], using the PySHARC driver [42], which enables efficient in‐memory communication between a Python interface and the SHARC routines.

### 3.1 Linear Vibronic Coupling Model

Using the results from the DFT calculations, the LVC model was parameterized following the procedure implemented in SHARC [42, 44]. In this model, the potential energy surfaces (PESs) of the relevant singlet and triplet states are represented as a linear expansion around the ground‐state PES $V_{0}$, using first‐order vibronic coupling terms W in the basis of mass‐ and frequency‐scaled normal mode coordinates Q

(1) $$V\left(Q\right)=V_{0}\left(Q\right)1+W\left(Q\right).$$

The ground‐state PES is approximated as a harmonic oscillator with frequency $\omega_{i}$,

(2) $$V_{0}\left(Q\right)=\sum_{i=1}^{3N-6}\frac{\hbar \omega_{i}}{2}Q_{i}^{2},$$

and the coupling terms are written as

(3) $$W_{nm}\left(Q\right)=\left\{\begin{array}{cc} \varepsilon_{n}+\sum_{i=1}^{3N-6}\kappa_{i}^{n}Q_{i}, & \text{if}\;n=m\\ \sum_{i=1}^{3N-6}\lambda_{i}^{nm}Q_{i}, & \text{if}\;n\neq m, \end{array}\right.$$

where $\varepsilon_{n}$ are the vertical excitation energies at the ground‐state optimized geometry, $\kappa_{i}^{n}$ and $\lambda_{i}^{nm}$ are the intrastate and interstate coupling terms, respectively, for the normal mode coordinate $Q_{i}$. These terms were obtained numerically from TDDFT calculations, using ORCA software, on geometries displaced by 0.05 AA from the ground‐state equilibrium geometry for each normal mode for both positive and negative directions. The SOCs in the LVC model potential were approximated by the SOCs obtained at the ground‐state equilibrium geometry. The PES parameterization was made considering three singlet and three triplet states. Parameters of the LVC are available in Section S1.

This approach has been extensively benchmarked against experimental ISC rates and triplet yields, demonstrating reliable predictive performance across systems with both efficient and inefficient ISC [42, 44, 47, 48, 49, 50]. Accordingly, LVC‐based TSH simulations are widely used to capture the key mechanistic factors governing ISC dynamics, also by direct comparison with experiments.

## 4 Results and Discussion

### 4.1 Heavy‐Atom Effect

The incorporation of heavier chalcogen atoms (S, Se, and Te) increases the SOC magnitudes between singlet and triplet excited states in organic molecules, a phenomenon called the heavy‐atom effect, thereby facilitating ISC between these states. As the atomic mass increases along the chalcogen series, the SOC matrix elements rise significantly, promoting faster population transfer to triplet states and leading to markedly shorter ISC time constants.

Table 1 shows the computed SOC magnitudes for the BD derivative with S, Se, and Te heteroatom substitutions. The data in this table demonstrate an increase in the SOC matrix elements along the series S → Se → Te; for example, the coupling $\left\langle S_{1}|H_{\text{SO}}|T_{3}\right\rangle$ increases from 0.06 (BD‐S) to 36.07 ${\mathrm{cm}}^{-1}$ (BD–Se), and then to 490.80 ${\mathrm{cm}}^{-1}$ (BD–Te). The same trend is observed in other relevant channels. However, for the couplings involving $S_{2}$, such as $\left|\langle S_{2}|H_{\text{SO}}|T_{3}\rangle \right|$, the value for BD–Se (259.27 ${\mathrm{cm}}^{-1}$) is higher than for BD–Te (62.14 ${\mathrm{cm}}^{-1}$). This result does not invalidate the heavy atom effect, but shows that there are other factors modulating the magnitude of the matrix elements, as the symmetry selection rules and the orbital superposition. Taking into account the $C_{2v}$ symmetry of the three molecules, we verified that the symmetry selection rules can not explain the small value of $\left|\langle S_{2}|H_{\text{SO}}|T_{3}\rangle \right|$ for BD–Te, as compared to BD–Se. However, when analyzing the matrix elements between the occupied orbitals of the $S_{2}$ and $T_{3}$ states, it is verified that the coupling for the BD–Se is intra‐configurational (the same orbital), while it is inter‐configurational for Bd–Te, with the occupied orbital of the $S_{2}$ state having no contribution on the Te atom. It results in much smaller superpositions between the occupied orbitals and a much smaller matrix element for BD–Te. The description of the symmetry of the different states and their respective characteristic orbitals is shown in Section S2.

**TABLE 1:** SOC magnitudes $\left\langle S_{n}|H_{\text{SO}}|T_{m}\right\rangle$, in ${\text{cm}}^{-1}$.

Molecule	$\left\langle S_{1}|H_{\text{SO}}|T_{1}\right\rangle$	$\left\langle S_{1}|H_{\text{SO}}|T_{2}\right\rangle$	$\left\langle S_{1}|H_{\text{SO}}|T_{3}\right\rangle$	$\left\langle S_{2}|H_{\text{SO}}|T_{1}\right\rangle$	$\left\langle S_{2}|H_{\text{SO}}|T_{2}\right\rangle$	$\left\langle S_{2}|H_{\text{SO}}|T_{3}\right\rangle$
BD–S	0.00	0.19	0.06	0.07	0.00	0.28
BD–Se	0.04	0.93	36.07	0.54	0.79	259.27
BD–Te	1.25	8.82	490.80	3.59	0.09	62.14

Abbreviations: BD, benzoxadiazole; SOC, spin–orbit coupling.

Notably, the direct $S_{1}\to T_{1}$ couplings ($\left\langle S_{1}|H_{\text{SO}}|T_{1}\right\rangle$) remain weak or null across all compounds, indicating that this ISC channel is inefficient. The most probable deactivation mechanism, therefore, involves a preferential ISC from $S_{1}$ or $S_{2}$ to higher triplet states ($T_{2}$ and/or $T_{3}$), which exhibit much stronger couplings, followed by a subsequent internal conversion to the $T_{1}$ state.

**FIGURE 2:**
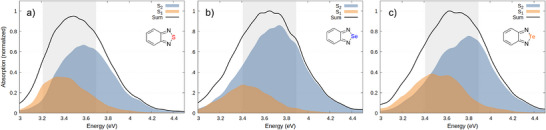
Absorption spectra of (a) BD–S, (b) BD–Se, and (c) BD–Te computed from a Wigner distribution of 1000 geometries, using a full width at half maximum (FWHM) of 0.1 eV for each excitation at each geometry. The contributing excited states, $S_{1}$ and $S_{2}$, are color‐labeled as indicated. The gray‐shaded area represents the excitation energy window from which the trajectories were initiated for the dynamics.

Figure 2 shows the calculated absorption spectra generated from calculating vertical $S_{1}$ and $S_{2}$ excitations of 1000 geometries selected from a Wigner probability distribution. The gray‐shaded region represents the 0.5 eV excitation window considered to later select the 500 initial conditions for the dynamics, chosen to encompass the main absorption peaks and the contributions from both $S_{1}$ and $S_{2}$ states. From the absorption spectrum, we can notice that both states can be populated with $S_{1}$ maximum centred at 3.4 eV for all the compounds, while the maxima of $S_{2}$, and of the total spectra, range between 3.5 and 3.8 eV and with slightly different relative intensities.

**FIGURE 3:**
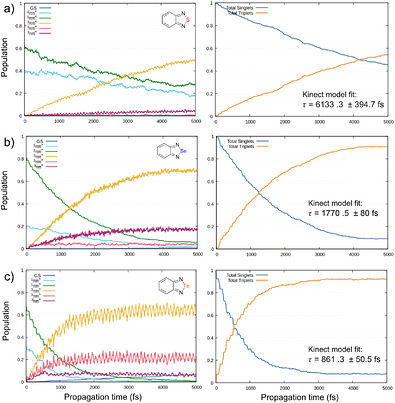
Time evolution of normalized quantum amplitudes of diabatic populations of three singlet and three triplet states (left panel) and normalized total populations of all of the singlet (blue curve) and triplet states (orange curve) summed over all the trajectories over 5000 fs of (a) BD–S, (b)BD–Se and (c) BD–Te.

Both states are ππ∗ states (See Section S2), and they are both likely to be populated in the selected excitation window, so we decided to include both of them as potential states on which the dynamics is initialized. 500 initial conditions within the selected range in the spectrum were propagated for 5000 fs and initialized on $S_{1}$ or $S_{2}$ based on their oscillator strength at the selected geometry Figure 3 shows the diabatic populations for the two singlet 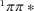 states and the two triplet 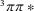 states and the triplet 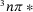 state (left panel), as well as the sum of the populations for all singlet and triplet states (right panel) of the BD series of molecules. The ISC can be observed by the increase in the electronic populations in the triplet states and a corresponding decrease in the populations of singlet states. These results clearly demonstrate that the progressive substitution S → Se → Te leads to a faster population transfer from the singlet to the triplet states, indicating a significant increase in the ISC rate, as a consequence of the heavy‐atom effect. Heavier atoms, with larger atomic numbers, exhibit stronger spin‐orbit coupling, which facilitates transitions between states of different spin multiplicities. It is highly encouraging for the design purposes of BD for PDT, that substituting the with Se and Te does not close the ISC pathway or promote other mechanisms but rather makes faster and more efficient the populations of the triplet states. Indeed, the kinetic analysis obtained by fitting the summed population of singlets and triplets shows a drastic speed up of the ISC, going from 6ps for BDS to less than 0.9 ps for BD–Te (Figure 3, right column).

The individual plots of electronic populations (Figure 3) also show that as the atom becomes heavier, the oscillation and redistribution among the triplet states become more pronounced, suggesting a more efficient coupling between triplet states due to the stronger relativistic contribution.

**FIGURE 4:**
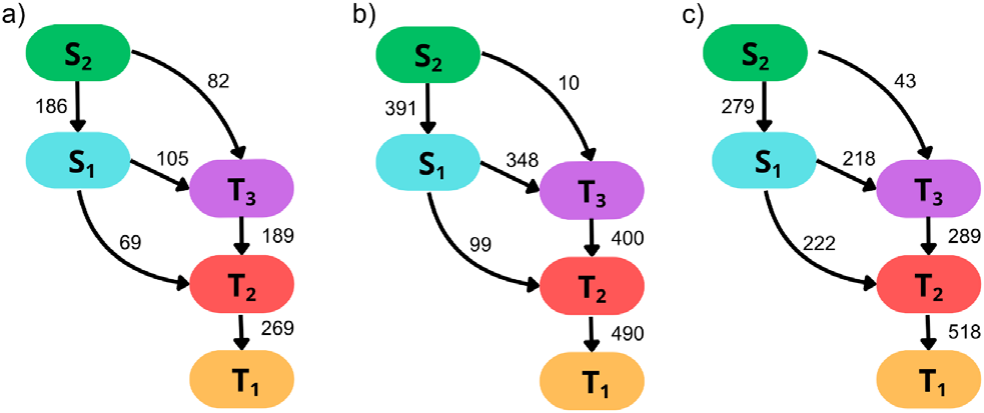
Number of net hops to the mains channels of ISC to (a) BD‐S, (b) BD‐Se, and (c) BD‐Te.

For a quantitative assessment of the population evolution and the ISC pathways, we analysed the net number of hops (Figure 4) and fit the population data to obtain a kinetic reaction model, as depicted in Figure 3 in the right panel. By globally fitting the time laws derived from this model, comprising the relevant singlet and triplet species, to the population data, we obtained the decay time constants. The associated statistical errors were computed using the bootstrapping method based on 100 resamples, representing the uncertainty inherent to the finite ensemble size. The plot of the kinetic model fit for all the molecules in Figure S1 is available in Section S4. The S‐substituted derivative displays the longest lifetime, with τ=6133.3±394.7 fs. In contrast, the Se‐substituted system shows a significant acceleration, yielding a lifetime of τ=1770.5±80 fs, which corresponds to approximately 3.5 times faster compared to the sulfur analog. The tellurium derivative exhibits the fastest dynamics with τ=861.3±50.5 fs, representing a substantial ≈7.1 times faster than the relative to the S‐compound. This monotonic decrease in lifetimes correlates directly with the increased spin–orbit coupling strength of the heavier chalcogens, efficiently facilitating the spin‐forbidden transition.

The analysis of the net hops between the excited state PES indicates the main transition channels between the states, either through IC or ISC. Figure 4 shows the main channels of the dynamics of excited states in the BD derivatives. BD–S derivative suggests a bifurcated decay mechanism originating from the $S_{2}$ state (280 net hops out). The $S_{2}$ state decays via two competing pathways: (i) efficient IC to the $S_{1}$ state (186 net hops), and (ii) direct ISC to the triplet manifold, primarily populating $T_{3}$ (82 net hops) and $T_{2}$ (12 net hops). The $S_{1}$ state, once populated, also undergoes rapid ISC to the states $T_{3}$ (105 net hops) and $T_{2}$ (69 net hops). Within the triplet manifold, a fast IC relaxation occurs, with population flowing from $T_{3}$ to $T_{2}$ (189 net hops) and subsequently from $T_{2}$ to $T_{1}$ (269 net hops).

The dynamics of BD–Se derivative reveal a dominant population flow following the pathway: $S_{2}\to S_{1}\to T_{3}/T_{2}\to T_{1}\to S_{0}$. The dynamics, primarily initiating from the $S_{2}$ state (402 net hops out), proceeds via IC to the $S_{1}$ state (391 net hops). From $S_{1}$, it evolves (via ISC) populating both the $T_{3}$ (348 net hops) and $T_{2}$ (99 net hops) states. Within the triplet manifold, rapid IC processes occur, from $T_{3}$ to $T_{2}$ (400 net hops) and subsequently from $T_{2}$ to $T_{1}$ (490 net hops). This leads to the accumulation of the electronic population in the $T_{1}$ state. Additionally, very few decays (four net hops) occur from $T_{1}$ to the $S_{0}$ ground state within the simulation timeframe.

Finally, BD–Te dynamics suggests a complex mechanism originating from the $S_{2}$ (334 net hops out) and $S_{1}$ (159 net hops out) states. The primary event is an efficient IC from $S_{2}$ to $S_{1}$ (279 net hops). This is followed by ISC from the $S_{1}$ state, which bifurcates to populate both $T_{2}$ (222 net hops) and $T_{3}$ (218 net hops). A minor direct ISC channel from $S_{2}$ to $T_{3}$ (43 hops) and $T_{2}$ (13 hops) is also observed. Once in the triplet manifold, the population undergoes rapid sequential IC relaxation: first from $T_{3}$ to $T_{2}$ (289 net hops), and then from $T_{2}$ to $T_{1}$ (518 net hops). The $T_{1}$ state acts as the dominant population, which subsequently decays back to the $S_{0}$ ground state (35 net hops in).

Overall, the analysis shows that the progressive substitution of S → Se → Te enhances the efficiency of intersystem crossing in the BD derivatives. Heavier chalcogens strengthen the SOC coupling and can greatly accelerate population transfer into the triplet manifold, resulting in markedly shorter ISC time constants and more complex triplet‐state redistribution.

### 4.2 D–A, D–D, and A–A Systems

As mentioned in the Introduction section, the push–pull framework of these molecules arises from the combination of an electron‐donating amine and an electron‐accepting nitro group, creating a D–A system (Figure 1). Replacing one of these groups, for example, substituting the acceptor by another donor (“push–push”, D–D), or the donor by another acceptor (“pull–pull”, A–A) can change the absorption and emission profiles. Although selenium in the benzoselenadiazole core would still promote intersystem crossing through the heavy‐atom effect, the excited‐state landscape and ROS‐forming capability would depend strongly on how such D–D or A–A substitutions modify the relative energies and characters of the singlet and triplet states [15]. The NBT1 and NBS1 systems were used as primary models for nonadiabatic dynamics simulations, and their behavior was compared to the two additional architectures, a pull–pull system with two nitro substituents and a push–push system with two amine substituents on the benzene ring (Figure 1). These variations enable a systematic assessment of how different D–A patterns influence the dynamics.

Parameterization using LVC models relies on a harmonic approximation of the potentials; therefore, normal modes associated with strongly anharmonic or periodic motions can lead to an inadequate description of the excited‐state potential energy surfaces [46]. For this reason, in the NBT3 and NBS3 systems, the normal modes related to torsional motions of the ligand around the BD core were excluded to avoid inconsistent parameterization. Note that no normal modes were removed during the PES parameterization of the other systems.

**FIGURE 5:**
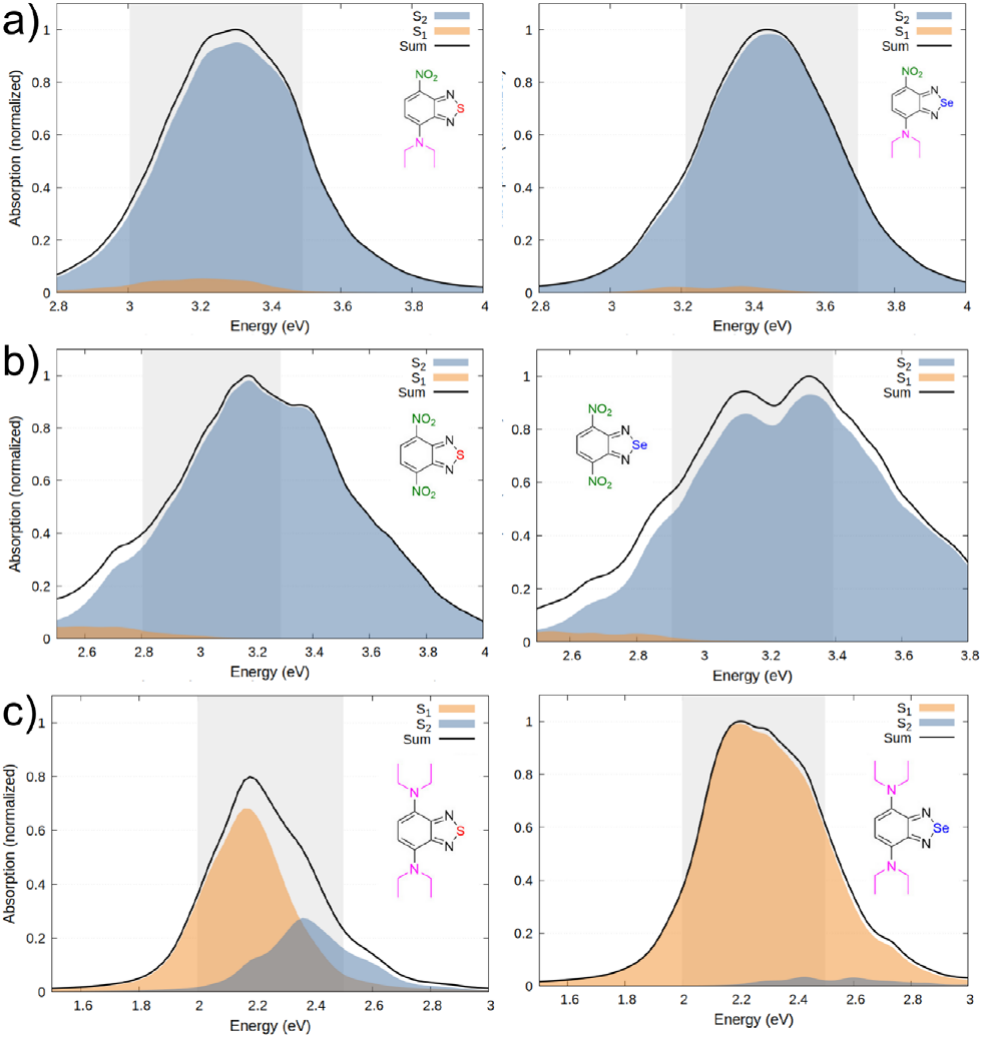
Absorption spectra of (a) NBT1 (left) and NBS1 (right), (b) NBT2 (left) and NBS2 (right), and (c) NBT3 (left) and NBS3 (right) computed from a Wigner distribution of 1000 geometries, using a full width at half maximum (FWHM) of 0.1 eV for each excitation at each geometry. The contributing excited states, $S_{1}$ and $S_{2}$, are color‐labeled as indicated. The gray‐shaded area represents the excitation energy window from which the trajectories were initiated for the dynamics.

Figure 5 shows the absorption spectra generated from a Wigner distribution of 1000 initial geometries, including the excitation energies of the states $S_{1}$ and $S_{2}$, as well as the energy window from which 500 initial conditions were selected to start the nonadiabatic dynamics. This window was chosen to encompass the main absorption peaks and the contributions from both excited states. In general, for NBT1 and NBS1 molecules (Figure 5a, push–pull systems) and NBT2 and NBS2 (Figure 5b, pull–pull systems), the spectra exhibit a stronger contribution from the $S_{2}$ state, with higher oscillator strengths, leading most trajectories to start in this excited state. In contrast, for NBT3 and NBS3 molecules (Figure 5c, push–push systems), the state $S_{1}$ contributes much more strongly to the first absorption band. In the absence of a strong electron acceptor, the system can lose its dominant ICT character [15, 19], causing most trajectories in dynamics to begin in the first excited state.

**FIGURE 6:**
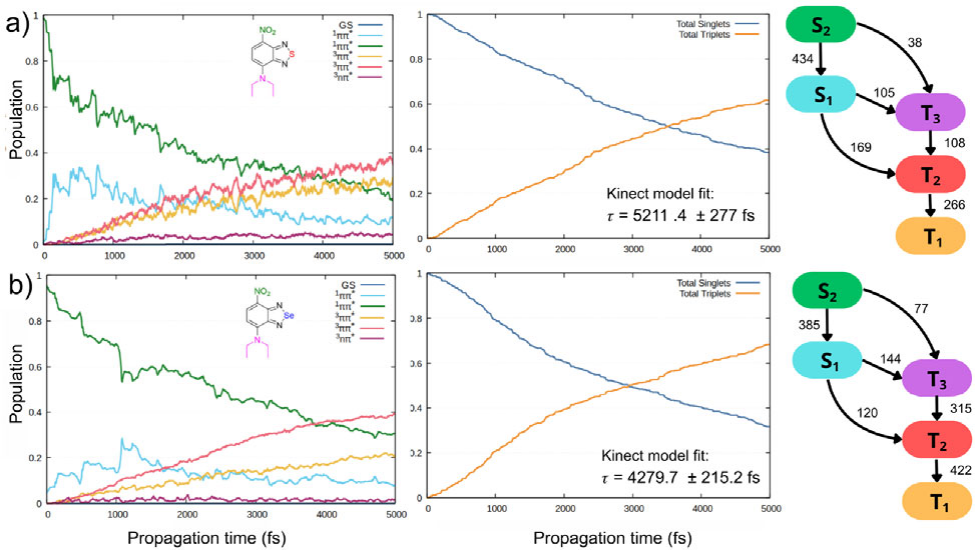
Time evolution of normalized quantum amplitudes of diabatic populations of three singlet and three triplet states (left panel) and normalized total populations of all of the singlet (blue curve) and triplet states (orange curve) summed over 5000 fs of (a) NBT1 and (b) NBS1.

As shown in Figure 6, the statistics over all 500 trajectories reveal that ISC occurs in both NBT1 and NBS1. The population of the $T_{2}$ state is comparable for the two systems (τ≈3900); however, the $T_{1}$ and $T_{3}$ states contribute more significantly to the photodynamics in NBS1, reducing the ISC time constant for NBS1 (τ≈2900 fs), compared to NBT1 (τ≈3600 fs), as reflected in the summed contributions of the singlet and triplet populations (central panel). The kinetic model fit reveals a time constants of τ=5211.4±277 fs and τ=4279.5±215.2 fs for NBT1 and NBS1, respectively. The plot of the fitting model can be verified in the Section S4.

Analyzing the number of hops (right panel in Figure 6) from the transition matrix, one can see that in the NBT1 system, the decay is dominated by IC from $S_{2}$ to $S_{1}$ (434 net hops), with a minor direct ISC channel to $T_{3}$ (38 net hops). The $S_{1}$ state acts as a significant bottleneck, retaining a large population (160 net hops) despite the ISC pathways to $T_{2}$ and $T_{3}$ that will lead to a final $T_{1}$ population, after IC processes inside the triplet manifold. In contrast, the NBS1 system exhibits an enhancement in ISC efficiency. The direct ISC channel from $S_{2}$ to $T_{3}$ doubles in magnitude (77 net hops), reducing the relative weight of the $S_{2}\to S_{1}$ IC pathway (385 net hops). Furthermore, the population retention in the $S_{1}$ state is reduced, indicating more efficient population transfer via ISC.

A comparison between the photodynamics of the NBT1 and NBS1 systems reveals that while the preferred relaxation pathway for both is the cascade $S_{2}\to S_{1}\to T_{n}\to T_{1}$, the efficiency of this channel can be modulated by the substituent. In the NBT1, the dynamics is characterized by a “bottleneck” effect at the $S_{1}$ state. Although the initial $S_{2}\to S_{1}$ internal conversion is dominant, the subsequent ISC is insufficiently fast to drain the $S_{1}$ population. In contrast, the NBS1 system exhibits a more efficient flow along the preferred pathway. The heavy‐atom effect enhances the spin–orbit coupling, promoting a competing direct ISC channel ($S_{2}\to T_{3}$) and accelerating the $S_{1}\to T_{2,3}$ transition. Consequently, the Se‐substitution effectively “unblocks” the relaxation cascade, favoring the population of the target $T_{1}$ state.

**FIGURE 7:**
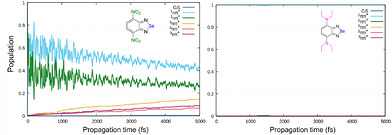
Time evolution of normalized quantum amplitudes of diabatic populations of 500 trajectories of three singlet and three triplet states over 5000 fs of NBS2 (left panel) and NBS3 (right panel).

Figure 7 shows the diabatic populations for the NBS2 and NBS3 molecules. It can be observed that neither system exhibits ISC within the 5000 fs simulation window. For the NBS2 molecule, the photodynamic process initiates with the population in the $S_{2}$ state, which decays rapidly and predominantly to the $S_{1}$ state via IC, right on the beginning of the dynamics. Upon reaching the $S_{1}$ state, the dynamics encounters a kinetic bottleneck, as the ISC channels to the triplet manifold are inefficient and slow compared to the rate of population inflow. Consequently, the vast majority of the excited population remains trapped in the $S_{1}$ state, favoring fluorescence emission over triplet state formation. The small fraction that successfully transitions to the triplet states relaxes in a cascade down to the $T_{1}$ state, but the final yield of this state is drastically limited by the unfavorable competition with the retention in the singlet state. In contrast, for the NBS3 molecule, the system, which starts predominantly in $S_{1}$, does not encounter any available IC or ISC pathways and therefore remains in the first excited state throughout the entire simulation. The results for NBT2 and NBT3 are similar, showing even lower population transfer to triplet states. The plot of diabatic populations of NBT2 and NBT3 are avaiable in the Supporting Information.

**FIGURE 8:**
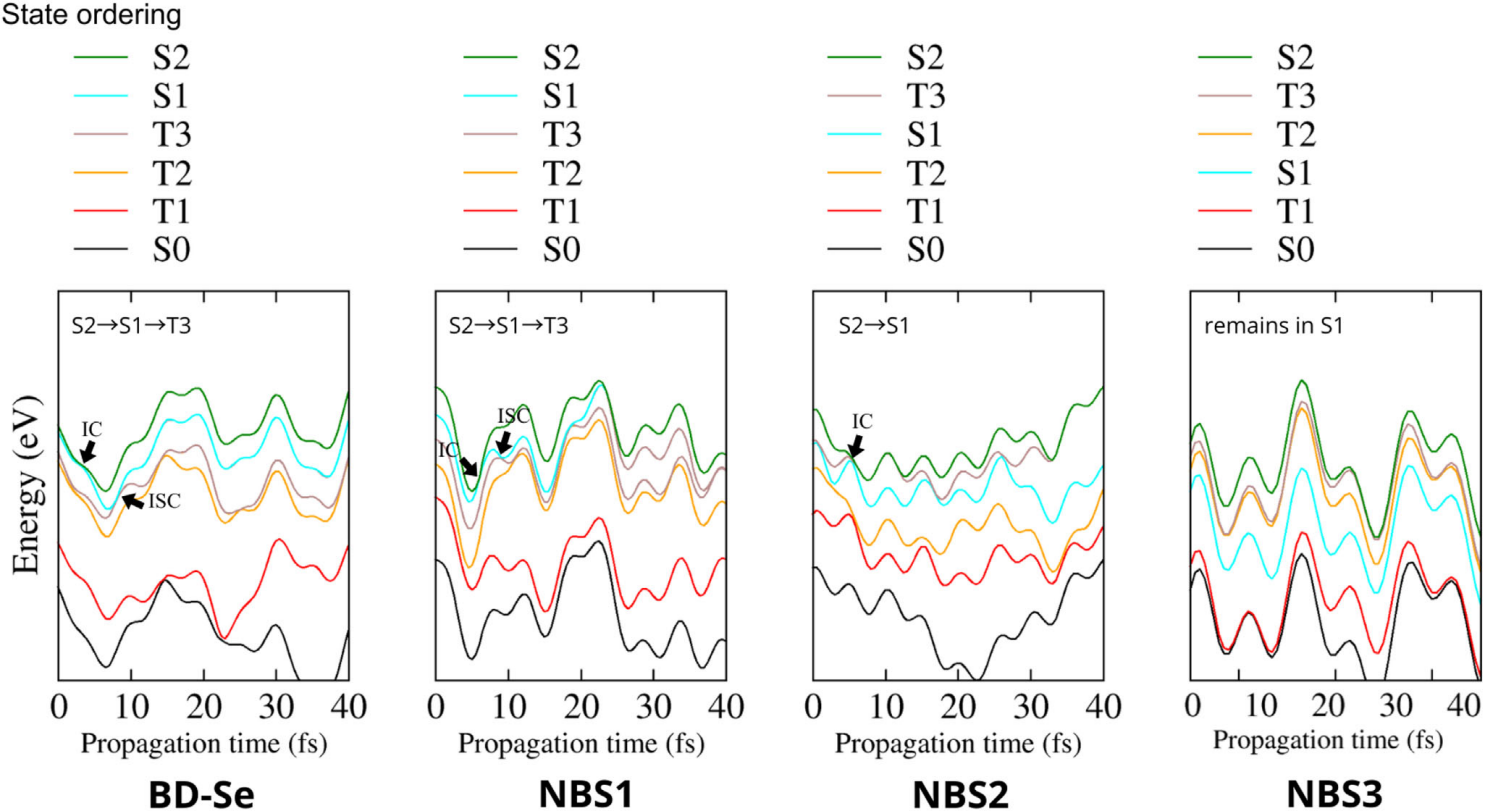
Selected trajectories to the molecules BD‐Se, NBS2, NBS2 and NBS3 with the ordering of excited states in the first 40 fs where IC and ISC occurs (arrows indicating) nd the sequence of active states.

Figure 8 shows some selected trajectories for the four systems studied. For BD‐Se and NBS1, we observe an IC from $S_{2}$ to $S_{1}$, followed by ISC to $T_{3}$, in agreement with the preferred ISC pathway identified previously ($S_{2}\to S_{1}\to T_{3}$). In the NBS2 molecule, a very fast IC into $S_{1}$ occurs within the first 10 fs. In contrast, for the NBS3 molecule, the dynamics starts in $S_{1}$ and no longer encounters conical intersections or energetically accessible states that would enable either IC or ISC. This behavior is more evident when the state ordering is verified. In NBS2, $S_{1}$ lies below $T_{3}$, while in NBS3, $S_{1}$ lies below $T_{3}$ and even $T_{2}$, effectively suppressing access to the preferred ISC channels.

Finally, it is important to note that, for NBS3, several normal modes associated with ligand‐core torsions were removed during the LVC parameterization of the excited‐state PES. This approximation may influence the results as any excluded mode could, in principle, mediate nonadiabatic coupling between these states. Therefore, this should be regarded as a methodological limitation of the LVC approach for this system.

## 5 Conclusion

We have presented a comprehensive study on the non‐adiabatic excited‐state dynamics of nitrobenzochalcogenadiazole derivatives, aimed at elucidating the mechanisms governing their potential as photosensitizers for PDT. By combining DFT calculations with surface hopping dynamics based on the LVC model, we provided a molecular‐level rationale for the interplay between heavy‐atom substitution and electronic “push–pull” effects. The nonadiabatic simulations reveal how charge‐transfer character, symmetry reduction, and vibronic coupling cooperatively shape the relaxation pathways, facilitating efficient population of triplet states.

Our simulations confirm that the heavy‐atom effect is the primary driving force for intersystem crossing in the benzochalcogenadiazole core. The substitution series S → Se → Te reveals a increase in spin–orbit coupling between singlet and triplet excited states, which directly correlates with the acceleration of the population transfer to the triplet manifold via a cascade mechanism ($S_{2}\to S_{1}\to T_{3/2}\to T_{1}$).

Crucially, however, this study demonstrates that heavy‐atom incorporation is not solely sufficient to guarantee efficient triplet generation. The comparison between different donor‐acceptor architectures reveals that the specific orbital topology governs the thermodynamic accessibility of the crossing points. While the canonical push–pull system (NBS1) maintains favorable state ordering for ISC, the disruption of this character in pull–pull (NBS2) and push–push (NBS3) analogues introduces severe kinetic bottlenecks. In these cases, the stabilization of the $S_{1}$ state below the relevant triplet manifolds effectively traps the excited population, suppressing ROS generation potential regardless of the selenium presence. This computational protocol proves to be a powerful tool for screening and guiding the rational engineering of next‐generation phototherapeutic agents by explicitly capturing the excited‐state dynamics that govern their photophysical performance.

## Conflicts of Interest

The authors declare that they have no potential conflict of interest.

## Supporting information


**Supporting File 1**: chem70941‐sup‐0001‐SuppMat.pdf

**Supporting File 1**: chem70941‐sup‐0002‐Data.zip
