# Supplementary material for: Design Principles for Next Generation of Small Organic Molecules for Photodynamics Therapy Revealed by Nonadiabatic Molecular Dynamics
Source: Chemistry. 2026 Mar 30;32(24):e70941. doi: 10.1002/chem.70941 (PMC13290423; doi:10.1002/chem.70941)
Supplement: Supplementary file 1 — Supporting File 1: chem70941‐sup‐0001‐SuppMat.pdf [file CHEM-32-e70941-s002.pdf]

Supplementary Materials

Design principles for next generation of small organic molecules for Photodynamics therapy revealed by nonadiabatic molecular dynamics

Vinícius N. Rocha*[a], Davide Avagliano*[b], Paulo C. Piquini[a]

[a] *Department of Physics, Federal University of Santa Maria, 97105-900, Santa Maria, RS, Brazil*
[b] *Ecole Nationale Supérieure de Chimie de ParisUniversité PSL, i-CLeHS, CNRS, Paris 75 005, France*

1. LVC.Template

The `LVC.template` file explicitly collects all constants of the Linear Vibronic Coupling model, diabatic energies (ϵ), spin–orbit couplings, intrastate linear vibronic constants (κ), interstate vibronic constants (λ), as well as charges and multipolar properties,and is used by SHARC to construct the LVC Hamiltonian employed in nonadiabatic dynamics simulations. More information about the code and the LVC.template can be obtained in the SHARC Manual (SHARC4.0 (2025). https://sharc-md.org/.)

The data supporting the findings of this study, including all Linear Vibronic Coupling (LVC) templates, are available in the public repository *ZENODO* at: https://doi.org/10.5281/zenodo.18237795

2. Symmetry of the BD-S, BD-Se, and BD-Te excited states and their respective orbitals participating in the spin-orbit coupling matrix elements

Table S1. Symmetry of the excited states of BD-S, BD-Se, and BD-Te. The molecules have C_{2v} point group symmetry

Excited State	BD-S	BD-Se	BD-Te
**S1**	B1	B1	A1
**S2**	A2	A2	B1
**S3**	A1	A1	B2
**T1**	B1	B1	A1
**T2**	A1	A1	B1
**T3**	A2	A2	A2

The excited states of BD-S, BD-Se, and BD-Te are obtained from the following electronic transitions between the canonical orbitals of the their respective electronic ground states

2.1 BD-S

- **S1**: 34 → 35 ; **S2**: 33 → 35 ; **S3**: 32 → 35
- **T1**: 34 → 35 ; **T2**: 33 → 35 ; **T3**: 32 → 35

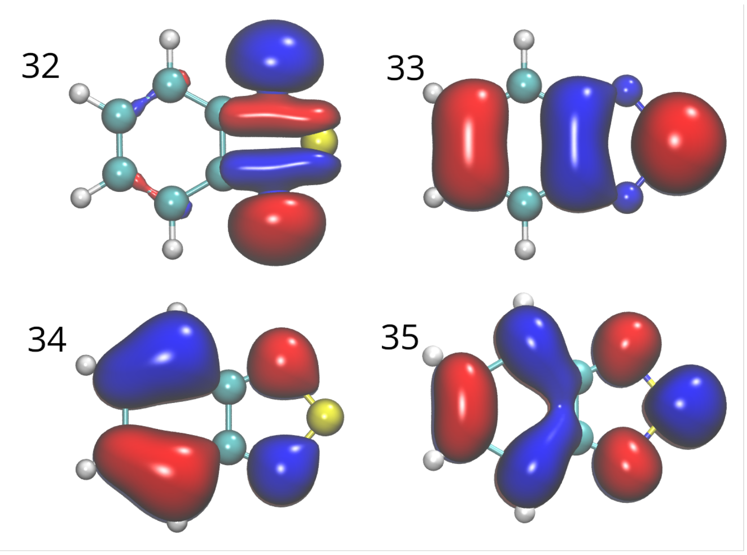

Figure S1. Canonical orbitals from the ground state of BD-S. The orbital nunmbered 34 is the canonical HOMO, and the 35 the LUMO.

2.2 BD-Se

- **S1**: 43 → 44 ; **S2**: 42 → 44 ; **S3**: 41 → 44
- **T1**: 43 → 44 ; **T2**: 42 → 44 ; **T3**: 41 → 44

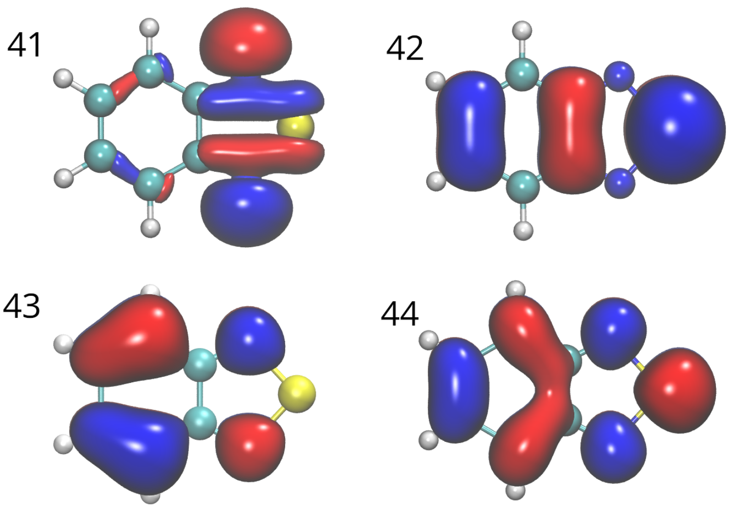

Figure S2. Canonical orbitals from the ground state of BD-Se. The orbital nunmbered 43 is the canonical HOMO, and the 44 the LUMO.

2.3 BD-Te

- **S1**: 52 → 53 ; **S2**: 51 → 53 ; **S3**: 50 → 53
- **T1**: 52 → 53 ; **T2**: 51 → 53 ; **T3**: 49 → 53

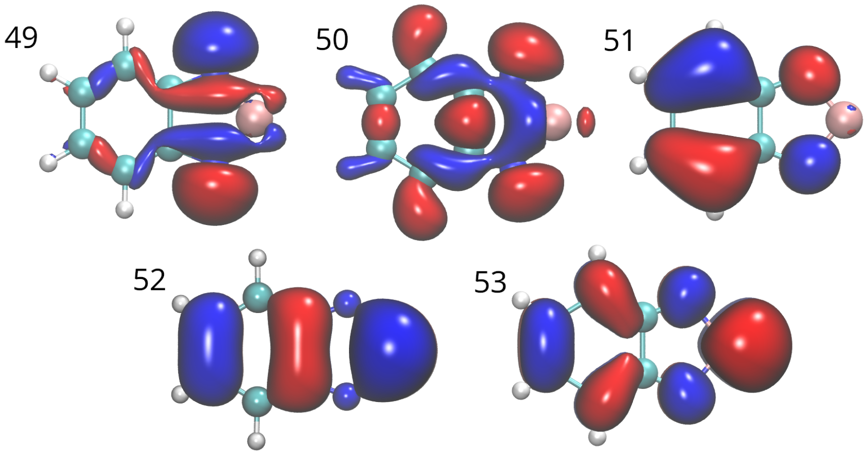

Figure S3. Canonical orbitals from the ground state of BD-Te. The orbital nunmbered 52 is the canonical HOMO, and the 53the LUMO.

3. Natural Transition Orbitals of the D-A, D-D and A-A systems

4. Fitted Time Constants

5. Diabatic Populations of NBT2 and NBT3

6. Benchmark TDDFT vs. LVC

The Figure 1 compares the time evolution of the electronic populations of BD-Se (Figure 1), over 5 trajectories, obtained using two distinct non-adiabatic dynamics schemes: (i) *surface hopping* (SH) coupled to a *Linear Vibronic*

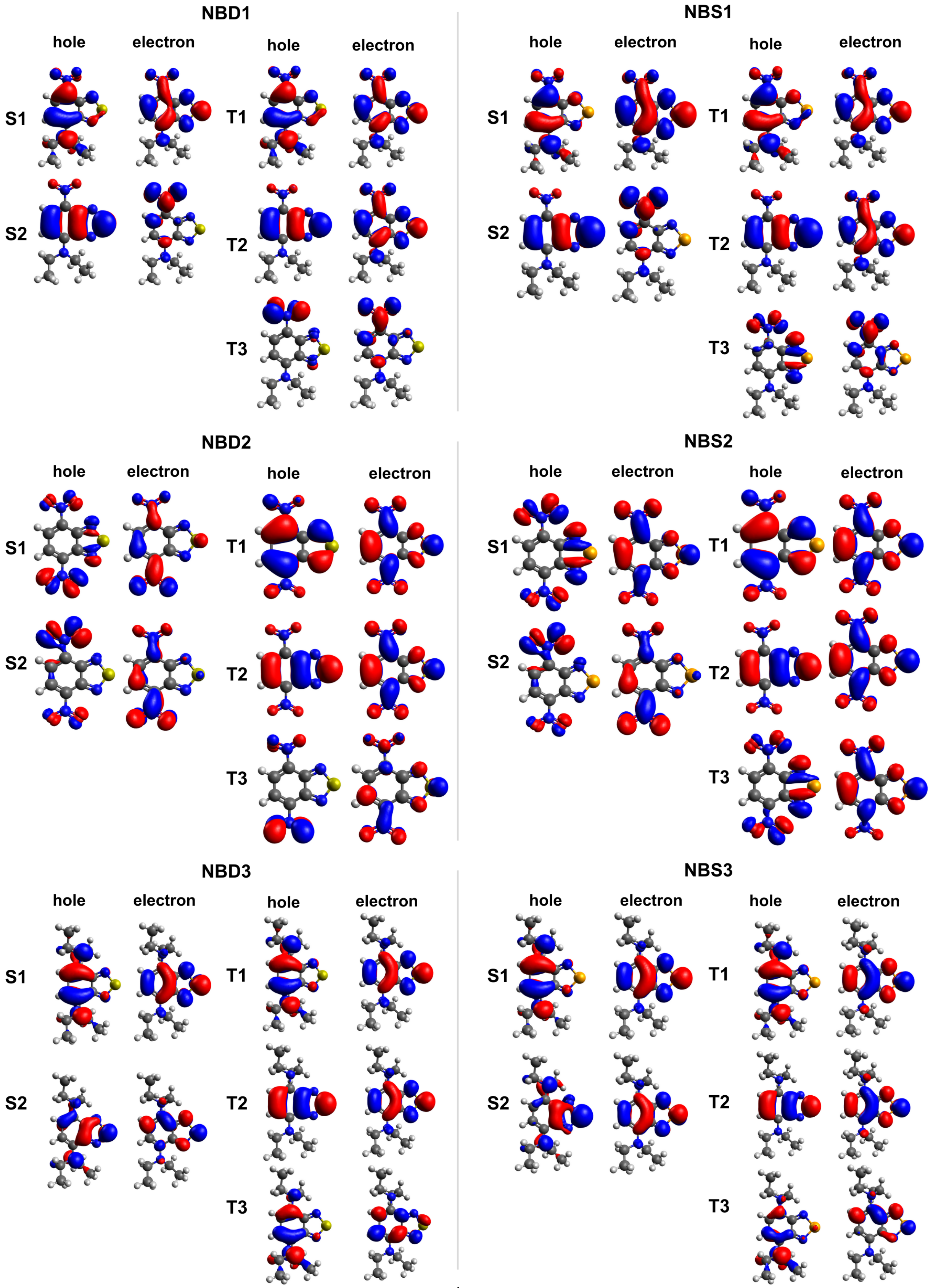

Figure S4. Natural Transition Orbitals of the D-A, D-D and A-A systems

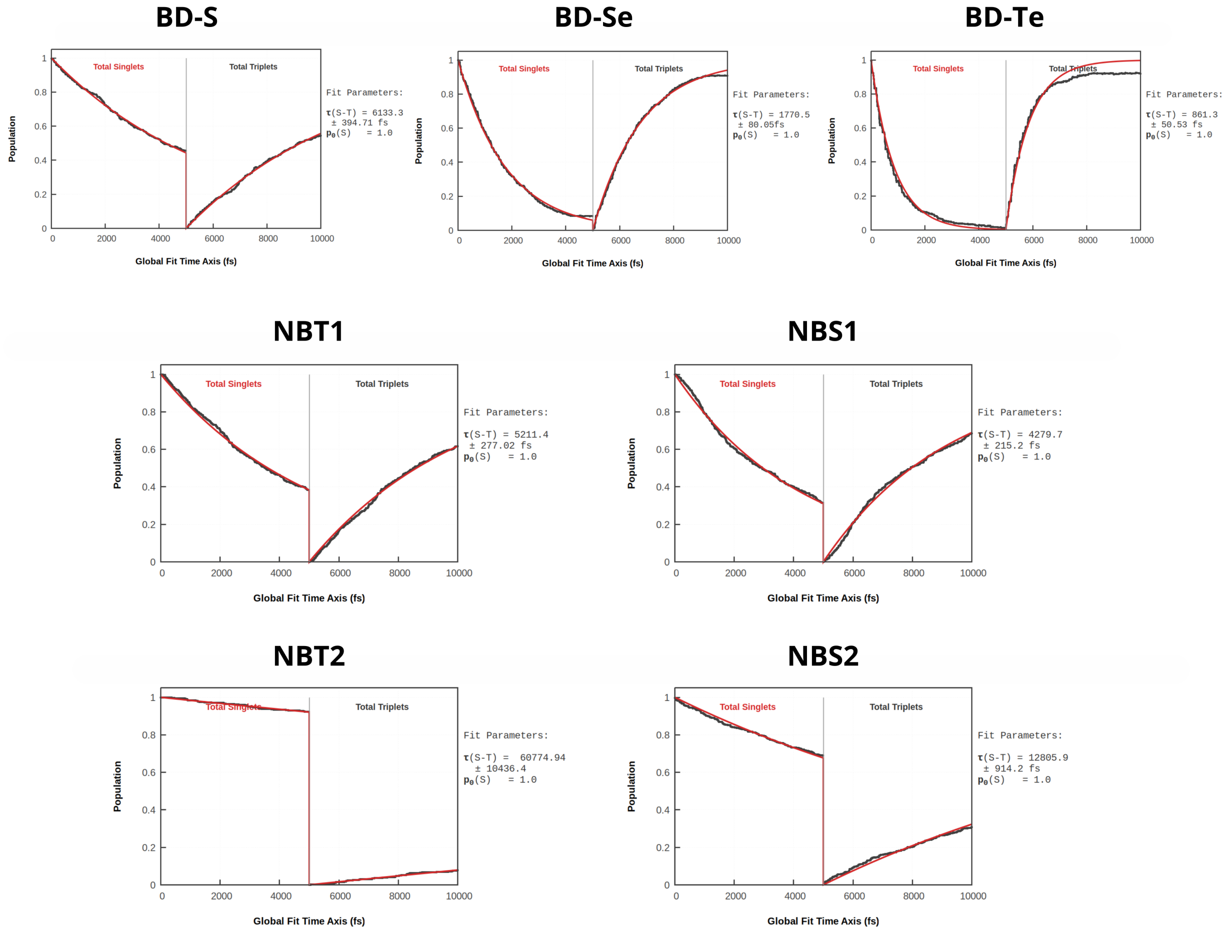

Figure S5. Evolution of diabatic populations, kinetic model and globally fitted time constants obtained from the population data.

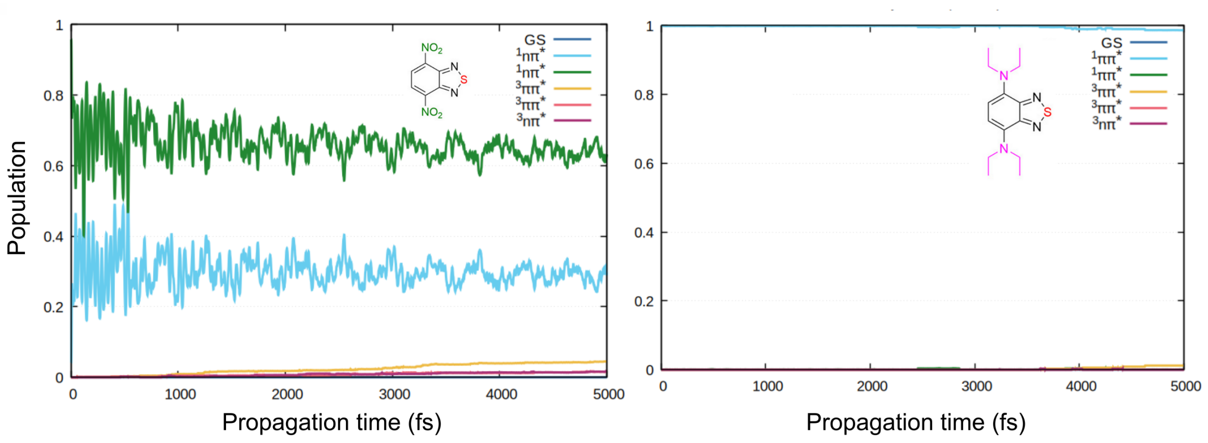

Figure S6. Time evolution of normalized quantum amplitudes of diabatic populations of 500 trajectories of 3 singlet and 3 triplet states over 5000 fs of NBT2 (left panel) and NBT3 (right panel).

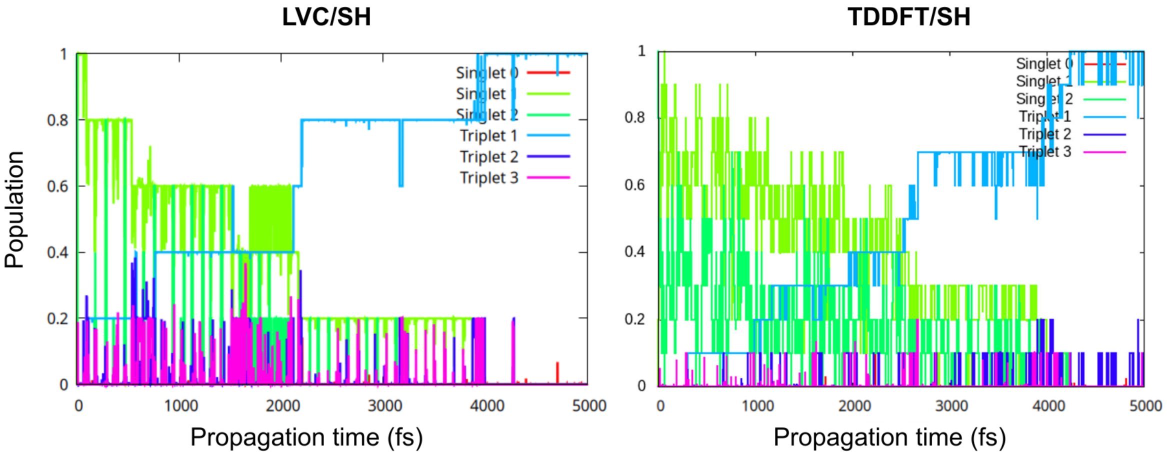

Figure 1: Time evolution of normalized populations in the MCH representation of 5 trajectories of 3 singlet and 3 triplet states over 5000 fs of BD-Se in the LVC/SH (left panel) and TDDFT/SH (right panel) level of theory.

fIGURE s7. Time evolution of normalized populations in the MCH representation of 5 trajectories of 3 singlet and 3 triplet states over 5000 fs of BD-Se in the LVC/SH (left panel) and TDDFT/SH (right panel) level of theory.

Coupling (LVC) model, in which the potential energy surfaces are parametrized prior to the dynamical simulation (LVC/SH, see 2. Computational Details in the manuscript), and (ii) *surface hopping* based on the *on-the-fly* evaluation of potential energy surfaces and couplings using TDDFT (TDDFT/SH). In both cases, five trajectories were propagated using exactly the same initial conditions, with geometries and velocities sampled from a Wigner distribution, thereby ensuring a direct comparison between the two methods. The results show excellent quantitative agreement. In particular, both approaches exhibit a progressive decrease in the populations of the singlet states, accompanied by a significant increase in the population of the lowest triplet state, T_1. For the sake of simplicity and consistency in the comparison, the MCH (*Molecular Coulomb Hamiltonian*) representation was adopted in both cases, as described in the SHARC manual.
